# Rapid and high-quality formation of dodecagonal quasicrystals and their approximants using a purely mechanical approach

**DOI:** 10.1093/nsr/nwag244

**Published:** 2026-04-24

**Authors:** Zhehua Jiang, Jianhua Zhang, Mengyuan Zhan, Jiaqi Si, Junchao Huang, Hua Tong, Ning Xu

**Affiliations:** Hefei National Research Center for Physical Sciences at the Microscale, University of Science and Technology of China, Hefei 230026, China; Department of Physics, University of Science and Technology of China, Hefei 230026, China; Department of Physics, University of Science and Technology of China, Hefei 230026, China; Department of Physics, University of Science and Technology of China, Hefei 230026, China; Department of Physics, University of Science and Technology of China, Hefei 230026, China; Hefei National Research Center for Physical Sciences at the Microscale, University of Science and Technology of China, Hefei 230026, China; Department of Physics, University of Science and Technology of China, Hefei 230026, China; Hefei National Research Center for Physical Sciences at the Microscale, University of Science and Technology of China, Hefei 230026, China; Department of Physics, University of Science and Technology of China, Hefei 230026, China; College of Physics, Guizhou University, Guiyang 550025, China

**Keywords:** dodecagonal quasicrystal, Archimedean tiling, formation and growth of quasicrystals

## Abstract

The conditions for forming quasicrystals and their approximants are stringent, normally requiring multiple length scales to stabilize the quasicrystalline order. Here, we report an unexpected finding that the approximants and motifs of dodecagonal quasicrystals can be spontaneously formed in the simplest system of identical hard disks, utilizing the unstable feature of the initial square packing subject to mechanical perturbations. Because only one length scale is involved, this finding advances our knowledge of quasicrystals and their approximants. Remarkably, applying this purely mechanical approach to systems known to form dodecagonal quasicrystals yields quasicrystalline order athermally and even single quasicrystals that exhibit the theoretical tiling patterns of perfect dodecagonal quasicrystals. Our study suggests a paradigm for the rapid synthesis of high-quality quasicrystals.

## Teaser

A mechanical approach is proposed that rapidly generates near-perfect or random dodecagonal quasicrystals by utilizing cooperative movements hidden in square lattice crystals. This approach also forms dodecagonal quasicrystal motifs and approximants in monodisperse hard-disk systems.

## INTRODUCTION

Quasicrystals, first discovered by Shechtman *et al.* [1], exhibit sharp Bragg peaks with symmetries forbidden in ordinary crystals and lack long-range translational order [2]. For the first two decades, quasicrystalline order was mainly observed in metallic alloys, until the discovery of quasicrystals in soft matter in 2004 [3]. The building blocks of soft matter may span multiple scales, making it a playground for diverse physical phenomena [4] and providing vast possibilities to tune interparticle interactions and the shape of structural units for the formation of quasicrystals [5–8]. Consequently, soft quasicrystals [9] have been observed in a wide range of soft matter systems, including dendrimer liquid crystals [3], star-shaped polymers [10], binary nanoparticle systems [11], block copolymer micelles [12], mesoporous silica [13], DNA-engineered biomolecules [14] and microspheres in applied magnetic and electric fields [15].

Compared to quasicrystals, their approximants, which share the same prototiles but exhibit long-range translational order, are easier to form. This makes quasicrystal approximants an ideal starting point for elucidating both the formation mechanism and structural attributes of quasicrystals [10,16–20]. Owing to their unique structural and physical properties, such as exceptional hardness, low friction and thermal stability, quasicrystals and their approximants hold promising applications in thermal insulation, surface coatings and advanced materials engineering [21].

Although the structures of quasicrystals and their approximants have been extensively studied [22], their formation mechanisms are not yet fully understood [23–25]. Typically, multiple characteristic length scales are involved in the structures of quasicrystals and their approximants. This leads to the prevailing hypothesis that their formation necessitates the introduction of multiple length scales. Consequently, studies often employ complex interparticle interactions with multiple length scales [6,9,26–30], polydisperse isotropic particles [31,32], or anisotropic particles with patches [33,34] or polyhedral shapes [24,35].

A notable exception to this general view was reported recently: both dodecagonal and octagonal quasicrystals can be formed by monodisperse isotropic disks interacting via simple spring-like repulsions [36,37]. In this case, quasicrystals are formed without explicitly engineering multiple length scales; instead, they rely on the high density of the system: the soft-core nature of the interactions promotes the self-assembly of disks into pentagons, which then serve as the fundamental building blocks for quasicrystal formation.

Note that disks interacting via spring-like repulsions effectively behave as hard ones at sufficiently low pressures [38,39]. Here, we address a more challenging question: can the simplest system of monodisperse hard disks spontaneously form quasicrystals or their approximants? Given the presence of only one length scale—the hard-disk diameter—this question may sound counterintuitive based on previous understanding. It is an indisputable fact that the thermodynamic equilibrium state of monodisperse hard disks is the hexagonal phase [40]. Although other metastable states, such as polycrystalline packings of hard disks, can be obtained via rapid compression from liquid states, to our knowledge, no spontaneous formation of complex crystalline or quasicrystalline structures has been reported, even under nonequilibrium conditions.

However, a straightforward analysis suggests that the idea might not be entirely infeasible. Theoretical tiling of a dodecagonal quasicrystal (DDQC) requires two building blocks: squares and equilateral triangles [41–43]. A hexagonal packing of hard disks is composed of equilateral triangles. Hard disks can also be arranged into a square lattice, which is, however, unstable due to the presence of soft modes, i.e. normal modes with zero frequency or energy. Therefore, these two building blocks can be constructed using hard disks, albeit at different packing densities. The critical challenge is to determine how to make these two building blocks spontaneously coexist and assemble into more complex motifs of quasicrystals or their approximants.

Furthermore, previous studies have shown that self-assembly normally leads to highly defective random-tiling quasicrystals or polyquasicrystals [14,24,27,44,45]. It thus remains challenging to form high-quality quasicrystals with fewer defects, particularly single quasicrystals. Theoretically, a perfect DDQC can be constructed by ideal square-triangle tiling following certain rules [41–43]. However, to our knowledge, single quasicrystals within this theoretical square-triangle tiling framework have rarely been achieved.

Utilizing the unstable feature of the square lattice, we unexpectedly observe the spontaneous formation of a DDQC approximant via a slight perturbation of the square packing of hard disks at constant pressure. By selectively removing pairs of disks from the square lattice, thus breaking the force balance, we observe the formation of two typical DDQC motifs. Although global formation of DDQCs has not yet been achieved with hard disks, this protocol significantly accelerates the formation of DDQCs in known DDQC-forming systems. Surprisingly, we achieve single DDQCs with square-triangle tiling. Our study reveals the previously unrecognized capability of hard disks to assemble into complex crystals and identifies a novel pathway for the formation of DDQCs and their approximants, providing a highly efficient and purely mechanical method to facilitate the formation of high-quality DDQCs.

## RESULTS

### Forming a DDQC approximant by hard disks

**Figure 1.**
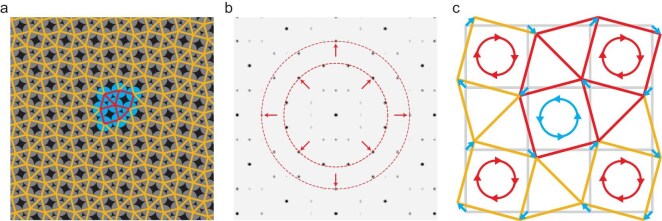
Formation of a DDQC approximant using hard disks and the transformation pathway. (a) DDQC approximant obtained from the perturbation of a square packing. Circles and lines represent disks and bonds, respectively. Red bonds highlight the $(3^2.4.3.4)$ Archimedean tiling. (b) Diffraction pattern of the approximant shown in (a). While 12-fold symmetry is visually apparent, close inspection reveals only four diffraction spots (marked by red arrows) lying on each red dashed circle, indicating a four-fold symmetry. (c) Local transformation pathway from a square packing to the approximant. Gray bonds represent the initial square packing. Orange and red bonds represent the structure of the resulting approximant. Red bonds highlight the periodic unit, i.e. the $(3^2.4.3.4)$ Archimedean tiling. Arrows indicate the displacements of disks during the transformation. Directional circles illustrate the rotational direction of the squares.

We first study static packings of disks interacting via harmonic repulsion (detailed in the Methods section below). At low pressures, these packings approximate hard-disk systems, as the harmonic repulsion effectively mimics hard-core exclusion. We begin with a square packing of disks at a pressure of $p=10^{-4}$. The square packing is unstable under any infinitesimal perturbation due to the presence of soft modes. Here, we introduce the perturbation by randomly displacing a disk away from its equilibrium position by a magnitude of 0.1 particle diameter, a value that falls within a regime where the resulting packing structure is robust against variations in the displacement magnitude. We have confirmed that other types of small perturbations yield equivalent results. After minimizing the enthalpy (detailed in the Methods section) at fixed pressure, we obtain a mechanically stable packing shown in Fig. 1a. By connecting contacting particles with bonds, the mixed tiling of squares and equilateral triangles can be clearly observed, demonstrating the spontaneous formation of a complex ordered structure.

As highlighted by the red bonds in Fig. 1a, a fundamental Archimedean tiling motif in our packing is ($3^2.4.3.4$), a prototile also prevalent in DDQCs. Figure 1b shows the diffraction pattern of the packing. Although it exhibits a 12-fold-like arrangement, as illustrated by the red dashed rings, only four diffraction spots lie exactly on each ring, suggesting a deviation from perfect 12-fold symmetry. Therefore, the packing is identified as a DDQC approximant with four-fold symmetry. The periodicity of the structure can be observed in Fig. 1a, and the number ratio of triangles to squares is 2, differing from $4/\sqrt{3}$ for perfect DDQCs [22,46]. All these characteristics indicate crystalline rather than quasicrystalline order. Nevertheless, it is rather unexpected that the simplest monodisperse hard disks can spontaneously form such complex crystals.

To illustrate the transformation from the square packing to the DDQC approximant, we show the displacement field in Fig. 1c. We focus on a local $4\times 4$ lattice region, which includes nine adjacent squares. The arrows represent disk movements, with the tail and head indicating the initial and final positions, respectively. The central square rotates either clockwise or counter-clockwise, while the four corner squares rotate in the opposite direction, creating a coordinated local rearrangement. Consequently, the four squares adjacent to the central square are squeezed into pairs of edge-sharing triangles.

As shown in the supplementary material, we apply the same protocol to a cubic packing of hard spheres. Similarly, owing to the instability of cubic packing, we obtain a three-dimensional packing with layered structures of DDQC approximants.

### Forming DDQC motifs by hard disks

**Figure 2.**
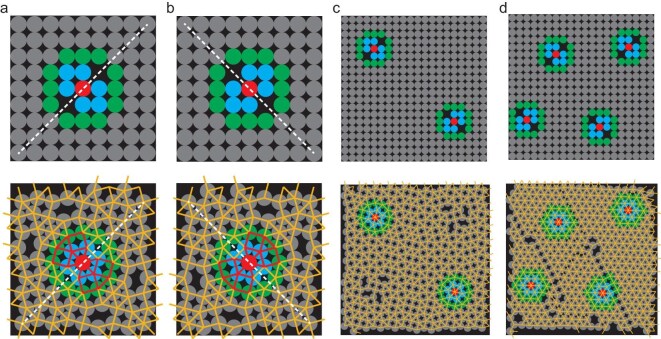
Formation of DDQC motifs using hard disks. (a) Introduction of a vacancy pair along the $45^{\circ }$ direction (dashed line) to a square packing of $N=81$ disks (top panel) and the resulting DDQC motif I (bottom panel). Disks transformed to the center, first shell and second shell of the motif are shown in red, blue and green, respectively. Solid lines in the bottom panel represent bonds, with red bonds highlighting two head-to-head $(3^2.4.3.4)$ Archimedean tilings. (b) Generation of DDQC motif II with a $30^{\circ }$ rotation relative to motif I in (a), achieved by removing a vacancy pair along the $-45^{\circ }$ direction (dashed line). (c and d) Introduction of 2 and 4 vacancy pairs, respectively, into an $N=400$ square packing (top panel) and the packing after the transformation (bottom panel).

The spontaneous formation of the DDQC approximant suggests an intriguing possibility of forming DDQCs using hard disks. We note that the fundamental motif of the square-triangle tiling of DDQCs is composed of 19 disks arranged in a specific geometry, as illustrated in the bottom panel of Fig. 2a. A central disk (red) is surrounded by two concentric polygons: an inner hexagon (blue) and an outer dodecagon (green). The square-triangle tessellation of this motif includes two head-to-head ($3^2.4.3.4$) Archimedean tilings. Based on the mechanism depicted in Fig. 1c, the formation of this pattern necessarily requires a local displacement field with mirror symmetry.

Figure 2a illustrates our strategy to induce particle motion with mirror symmetry. As shown in the top panel, we remove a pair of disks from the square packing, which are the second-nearest neighbors of a central disk (red) and align along the $45^{\circ }$ dashed line passing through the central disk. This removal disrupts the force balance on the eight disks immediately surrounding these two newly created vacancies, leading to particle rearrangements under constant pressure. The presence of these two vacancies induces mirror-symmetric movements of disks about the $45^{\circ }$ line. Following enthalpy minimization, a DDQC motif is successfully produced, as shown in the bottom panel of Fig. 2a. Surrounding the motif, various tiling patterns emerge, including the ($3^2.4.3.4$) tiling. Figure 2b demonstrates that removing a pair of disks along the $-45^{\circ }$ line results in another motif with a $30^{\circ }$ rotation relative to that in Fig. 2a. Both motifs are characteristic of the square-triangle tiling of perfect DDQCs. Since each motif alone produces only six-fold dihedral symmetry, their combination is essential to form the 12-fold symmetry in perfect DDQCs [43,47]. Therefore, our mechanical approach effectively generates both motifs, providing a potential pathway to construct DDQC structures. We denote the motifs in the bottom panels of Fig. 2a and b as motif I and motif II, respectively.

The question now becomes: can long-range DDQC order be realized by introducing a sufficient number of vacancy pairs into the square packing? We start the investigation by randomly introducing $n_{\rm v}$ pairs of both types of vacancies into the system and distributing them uniformly in space. As shown in Fig. 2c, for a square packing of $N=400$ hard disks, two DDQC motifs are generated when $n_{\rm v}=2$. However, when $n_{\rm v}= 4$, Fig. 2d indicates that our approach fails to generate or sustain DDQC motifs. Instead, hexagonal packing dominates after the transformation. As shown in the supplementary movie, during the early stage of the transformation, ($3^2.4.3.4$) tilings emerge and grow but are eventually overwhelmed by the rapid expansion of the hexagonal packing. For hard disks, hexagonal packing is thermodynamically stable. In contrast, DDQC approximants and structures with DDQC motifs are metastable, being much less stable than the hexagonal packing and susceptible to destabilization under large perturbations. Increasing the number of vacancy pairs enhances the perturbation, destabilizing these complex structures and driving the system toward the more stable hexagonal phase.

### Athermal formation of DDQC order in a DDQC-forming system

Although our current mechanical approach fails to generate global DDQC order using hard disks, the successful generation of DDQC motifs suggests a potential avenue for DDQC exploration. To test this feasibility, we apply the same approach to a system with a two-length-scale (TLS) interaction potential (detailed in the Methods section below). This system has been shown to self-assemble into a DDQC when equilibrated at appropriate densities and low temperatures [48–50]. We initialize the system by arranging TLS disks into a square lattice and randomly introducing $n_{\rm v}$ vacancy pairs ($n_{\rm v}/2$ for motif I and $n_{\rm v}/2$ for motif II, detailed in the Methods section). We then adjust the number density to $\rho =0.94$, at which DDQC self-assembly can occur. An energy minimization is then performed to obtain static packings at $T=0$.

**Figure 3.**
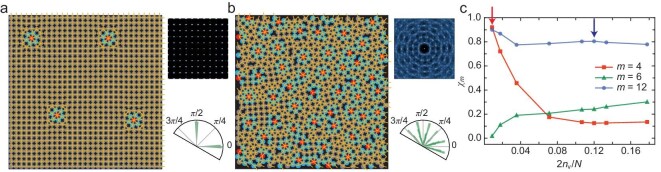
Formation of DDQCs using TLS disks through a mechanical approach. (a and b) Results for introducing $n_{\rm v}=4$ and 54 vacancy pairs, respectively, into an $N=900$ square packing, followed by energy minimization. The left panel shows the configuration with bonds. Disks in red and blue are those expected to become the center and first shell of DDQC motifs. The top-right and bottom-right panels show the diffraction pattern and the distribution of bond angles, respectively. (c) Evolution of the *m*-fold orientational order $\chi _m$ for the resultant packings with $2n_{\rm v}/ N$. The two arrows point to $n_{\rm v}=4$ and 54, as shown in (a) and (b), respectively.

As shown in the left panel of Fig. 3a, $n_{\rm v}=4$ vacancy pairs lead to four DDQC motifs in the static packing with initially $N=900$ TLS disks. Unlike hard-disk systems, the square-lattice structure is still maintained around the motifs. This preservation of square-lattice order is attributed to the long-range particle interactions. Each disk interacts not only with its nearest neighbors but also with distant neighbors, stabilizing the square-lattice structure. As a result, four-fold symmetry still dominates in the diffraction pattern, as confirmed in the top-right panel of Fig. 3a. The bottom-right panel of Fig. 3a illustrates the distribution of the bond angle $\theta$, with $\theta =0$ defined as the right horizontal direction. The bonds are predominantly aligned along the two principal directions of the square lattice, $\theta =0$ and $\pi /2$. As defined in Fig. 2a and b, motifs I and II contribute six characteristic DDQC angles: $-\pi /12$, $\pi /12$, $\pi /4$, $5\pi /12$, $7\pi /12$ and $3\pi /4$ [22,42,43]. Since there are only four motifs in Fig. 3a, their contribution to the bond-angle distribution is negligible.

Figure 3b shows the results for $n_{\rm v}=54$ vacancy pairs. Unlike the case of hard disks, the left panel illustrates that a significant fraction of DDQC motifs persists even at such a high concentration of vacancy pairs. Quasicrystalline order is evident in the diffraction pattern. Furthermore, the bond-angle distribution reveals well-defined peaks corresponding to the six characteristic DDQC angles. Thus, by simply introducing vacancy pairs into a square lattice, DDQC order is rapidly induced via an athermal approach.

Figure 3c illustrates the evolution of the average local structural orders with $2n_{\rm v}/N$ for $N=900$. Here, we present the results for 4-, 6- and 12-fold symmetries, denoted as $\chi _4$, $\chi _6$ and $\chi _{12}$, respectively (detailed in the Methods section below). For the initial square lattice ($n_{\rm v}=0$), both $\chi _4$ and $\chi _{12}$ equal 1, while $\chi _6$ is almost zero, reflecting the incompatibility of six-fold symmetry with the four-fold symmetry of the square lattice. As $n_{\rm v}$ increases, both $\chi _4$ and $\chi _{12}$ decrease and saturate at a plateau. However, $\chi _4$ decreases to a low value of ${\sim }0.1$, whereas $\chi _{12}$ saturates at a high value of ${\sim }0.8$, indicating the breaking of four-fold symmetry and the emergence of 12-fold symmetry. Meanwhile, $\chi _6$ gradually increases. Since hexagonal and square structures are fundamental elements in DDQCs, $\chi _4$ and $\chi _6$ remain nonzero but are relatively small in DDQCs. For the $n_{\rm v}=54$ case shown in Fig. 3b, $\chi _{12}\approx 0.80$, $\chi _4\approx 0.12$ and $\chi _6\approx 0.26$, demonstrating that the observed 12-fold symmetry is primarily due to DDQC order rather than mixed contributions from four- and six-fold symmetries.

### Enhancing DDQC order via thermal treatment

To quantify the quality of the DDQC order, we use the following metric. For a bond with angle $\theta$, we identify the closest of the six characteristic DDQC angles to it, denoted as $\theta _{\rm c}$, and calculate the deviation $\Delta \theta =|\theta -\theta _{\rm c}|$. Therefore, smaller values of $\Delta \theta$ indicate better DDQC orders.

**Figure 4.**
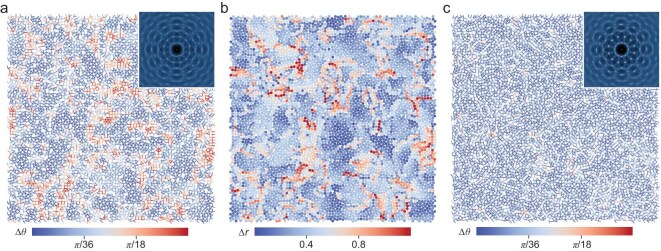
Enhancement of the DDQC order through thermal treatment for systems with TLS disks. (a) Spatial distribution of the deviation of bond angle from the characteristic DDQC angles, $\Delta \theta$, for a packing mechanically transformed from an $N=6400$ square packing with $n_{\rm v}=427$ vacancy pairs. The color bar indicates the magnitude of $\Delta \theta$. (b and c) Displacement field and spatial distribution of $\Delta \theta$, respectively, after equilibrating the configuration in (a) over a time duration of $t=5\times 10^{5}$ at $T=0.12$. The color bars in (b) and (c) indicate the magnitudes of particle displacement, $\Delta r$ and $\Delta \theta$, respectively. Diffraction patterns inserted in (a) and (c) highlight the growth of DDQC order after the thermal treatment.

Figure 4a presents a TLS disk packing obtained via our mechanical, athermal approach, where DDQC order is evident from the diffraction pattern. This packing is induced by introducing $n_{\rm v}=427$ vacancy pairs into an $N=6400$ square packing at $\rho =0.94$. The bond color reflects its $\Delta \theta$ value. In regions where vacancy pairs are introduced and DDQC motifs are formed, the bonds generally have small values of $\Delta \theta$, whereas other regions show relatively larger values of $\Delta \theta$. Therefore, while the mechanical approach successfully induces DDQC order, it is not sufficient to optimize the global order, indicating the necessity for additional treatments.

We thus heat the packing to a low temperature of $T=0.12$ and equilibrate it for a duration *t*. Figure 4b illustrates the spatial distribution of particle displacement over a duration of $t=5\times 10^5$, with the color coding indicating the magnitude of particle displacement, $\Delta r$. A strong spatial correlation between $\Delta r$ and $\Delta \theta$ can be observed by comparing Fig. 4a and b, with larger $\Delta r$ typically occurring in regions with relatively larger $\Delta \theta$. As illustrated in Fig. 4c, after equilibration, the majority of bonds exhibit small $\Delta \theta$ values, and the spatial distribution of $\Delta \theta$ becomes significantly more uniform. The optimization effect of the thermal treatment is further demonstrated by the sharpening of spots in the diffraction pattern.

### Efficiency of the mechanical approach

A notable advantage of the mechanical approach is the instantaneous development of DDQC order, significantly accelerating DDQC formation. As shown above, thermal treatment primarily serves to optimize regions with weak DDQC orders. It is thus expected that the overall process would be much less time-consuming than the direct formation of DDQCs from the liquid state at a specific temperature.

**Figure 5.**
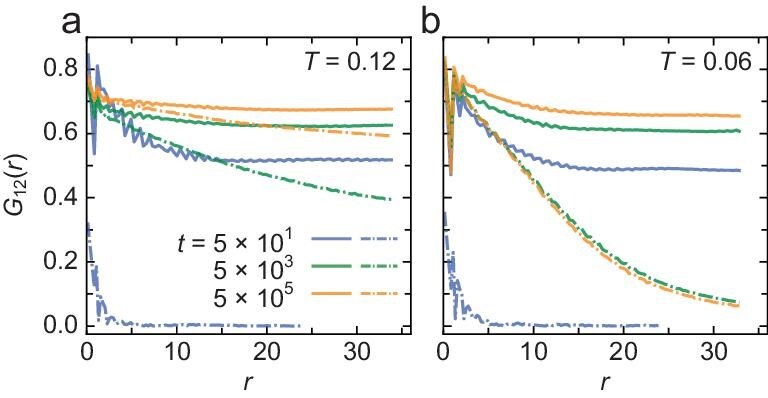
Comparison of DDQC growth in systems starting from the mechanical approach and a quenched liquid. (a and b) Time evolution of the correlation function of the 12-fold order parameter, $G_{12}(r)$, at $T=0.12$ and 0.06, respectively. Solid and dash–dot lines are for states from the mechanical approach and the liquid state, respectively. The legend indicates the specific instants used for comparison. The same TLS systems as in Fig. 4 are used.

In Fig. 5a, we compare the time evolution of the correlation function of the 12-fold bond-orientational order, $G_{12}(r)$ (detailed in the Methods section below), at $T=0.12$ for two initial conditions. Starting from a state generated by the mechanical approach, $G_{12}(r)$ exhibits a plateau at large distances *r*, indicating the presence of a long-range DDQC order. The plateau value increases over time, demonstrating the growth of the overall DDQC order. In contrast, starting from a liquid state quenched from a high temperature, $G_{12}(r)$ is small at short times. At longer times, $G_{12}(r)$ decays with increasing *r*, suggesting the absence of long-range order. With increasing time, $G_{12}(r)$ increases and becomes flatter, indicating the growth of DDQC order. However, it remains smaller than that from the mechanical approach. This comparison highlights the efficiency of the mechanical approach in facilitating the formation of DDQCs. For the same amount of time, the mechanical approach enables significantly faster formation of DDQCs with better global order.

Figure 5b further showcases the effectiveness of the mechanical approach. At a lower temperature ($T=0.06$), the growth of DDQC order from the liquid state is significantly hindered within the same time scale as in Fig. 5a. Parameter $G_{12}(r)$ evolves rather slowly and exhibits rapid decay with increasing *r* even at long times. In contrast, the time evolution of the state from the mechanical approach remains comparable to that at $T=0.12$. Therefore, at low temperatures, when the self-assembly of DDQCs from a liquid state is impeded by sluggish particle dynamics, the mechanical approach can still promote structural relaxation and the growth of DDQC order in a non-diffusive manner. In addition to facilitating the DDQC formation, the mechanical approach reveals a distinct pathway for DDQC growth.

Here we show results for two temperatures, both below the melting temperature $T_{\rm m}\approx 0.24$ (detailed in the supplementary material). In the supplementary material, we compare $G_{12}(r)$ for different temperatures, which demonstrates that $T_{\rm m}$ roughly marks the upper temperature limit for obtaining enhanced DDQC order via thermal treatment.

As discussed above, the thermal treatment is used to repair local DDQC order after the mechanical approach. In the supplementary material, we demonstrate that the thermal treatment can be replaced with small-amplitude oscillatory shear. The shear plays a similar role to temperature in exciting local particle motion and optimizing DDQC order. Combined with oscillatory shear, we enable the formation and optimization of DDQC order in a purely mechanical manner.

### Athermal formation of square-triangle-tiling single DDQCs

In previous sections, we introduced vacancy pairs randomly in space, resulting in random-tiling DDQCs. Direct self-assembly from liquid states likewise leads to random-tiling DDQCs within accessible simulation timescales. In fact, most previous studies have succeeded in obtaining highly defective random-tiling DDQCs or poly-DDQCs [14,15,24,27,44,45] that deviate from perfect square-triangle tiling. To date, no robust method exists for effectively synthesizing square-triangle-tiling single DDQCs.

Because of quasi-periodic symmetry, perfect quasicrystals exhibit self-similar features. For example, in the square-triangle tiling construction, if we treat motifs I and II (defined in Fig. 2a and b) as two first-order hyperparticles, these hyperparticles can assemble into larger hierarchical tilings, which in turn are second-order hyperparticles (i.e. larger motifs). By iterating this process, increasingly higher-order hyperparticles—and simultaneously perfect DDQCs—can be constructed. Given that the first-order hyperparticles can be generated by introducing vacancy pairs into a square lattice, can square-triangle-tiling single DDQCs be mechanically synthesized through the delicate introduction of vacancy pairs?

**Figure 6.**
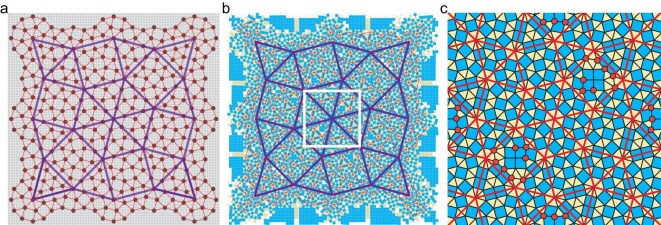
Formation of the square-triangle tiling using TLS disks through the mechanical approach. (a) Illustration of how to locate vacancy pairs in a square packing. The gray circles are TLS disks in a square packing. The black circles are centers of the first-order hyperparticles, connected by red lines. The ratio between the length of the red lines and the lattice constant of the square packing is 4.1. For each black circle, we identify the closest gray circle as the center of a vacancy pair to be removed, as detailed in the text. The purple lines connect centers of the second-order hyperparticles. The square packing initially contains $N=6400$ disks. (b) Square-triangle tiling of the resulting packing after minimizing the energy of the square packing with vacancy pairs at $\rho =0.94$. Squares and triangles are filled with blue and yellow, respectively. The red and purple lines connect centers of the first- and second-order hyperparticles, respectively. (c) Enlarged view of the white square region in (b). Red circles mark the topological point defects.

As illustrated by Fig. 6a, we overlay a perfect square-triangle tiling (black circles connected by red lines) on a square packing of TLS disks (gray circles). We refer the reader to the supplementary material for details of the theoretical construction of the square-triangle tiling. Each black circle represents the center of a first-order hyperparticle. We also show in Fig. 6a another tiling by connecting centers of the second-order hyperparticles with purple lines. These two sets of tiling explicitly exhibit self-similarity. For each black circle in Fig. 6a, we identify the closest gray circle (the black circle does not necessarily fall exactly on the gray one) as the center of a vacancy pair. We then remove a pair of disks (along either $45^{\circ }$ or $-45^{\circ }$ direction) according to the type of the corresponding first-order hyperparticle (motif I or II), determined by the self-similar nature of the perfect square-triangle tiling (detailed in the supplementary material). To achieve the optimal DDQC formation, we adjust the nearest spacing between black circles and find that the optimal value is approximately 4.1 times the original square-lattice constant.

Figure 6b shows the square-triangle tiling of the packing after energy minimization at $\rho =0.94$. We connect centers of the first-order (second-order) hyperparticles by red (purple) lines, which perfectly maintain the theoretical tiling, except for the boundary regions. An enlarged view of the white square region in Fig. 6b is shown in Fig. 6c. It exhibits near-perfect square-triangle tiling, except for isolated topological point defects (see the supplementary material for the definition) [51], which are marked by red circles in Fig. 6c. Analogous to point defects in ordinary crystalline lattices—which are local perturbations that do not destroy the global orientational order—these defects do not disturb the 12-fold symmetry of the ideal square-triangle tiling. Using our mechanical approach, we successfully produce large-scale single DDQCs that perfectly maintain the higher-order theoretical tilings, except for some point defects at the single-particle level.

## DISCUSSION

By perturbing the square packing of hard disks, we realize the spontaneous formation of a DDQC approximant and the local formation of DDQC motifs via a purely mechanical approach. We further demonstrate that this mechanical approach can facilitate the self-assembly of DDQCs in known DDQC-forming systems. Our approach shows high efficiency in forming robust DDQC order athermally, accelerating DDQC growth with the aid of thermal treatment and enabling DDQC growth even at rather low temperatures, when self-assembly of DDQCs from liquid states is significantly suppressed. Notably, our purely mechanical approach enables single DDQCs with isolated point defects to form spontaneously while preserving perfect higher-order square-triangle tilings—a challenge for conventional methods.

To our knowledge, the spontaneous formation of complex crystals and DDQC structures has not been previously anticipated in the simplest system of identical hard disks. Our findings thus advance our understanding of DDQCs and their approximants. Moreover, the non-diffusive growth of DDQC order triggered by our mechanical approach provides insights into the growth pathways of DDQCs. Our results can be validated in experimental systems, such as colloidal suspensions using optical tweezers to construct and manipulate the square packing. In practice, introducing vacancy pairs sequentially rather than simultaneously offers greater experimental feasibility, as confirmed by our simulations (see the supplementary material for details).

While our study focuses on DDQCs, it would be interesting to investigate whether quasicrystals with other symmetries can be generated using our mechanical approach. We expect that our approach may be effective for quasicrystals with squares as a tiling element, such as octagonal quasicrystals. However, for quasicrystals lacking squares as a tiling element, it remains unclear whether the mechanical approach would be effective with the design of alternative unstable initial structures.

Moreover, while we have realized DDQC formation in single-component systems, it remains an open question whether our mechanical approach can be extended to binary or multicomponent systems. Such an extension would be more relevant to metallic quasicrystals, which are themselves alloys.

## METHODS

### Models

Our systems are square cells with side length *L*, consisting of *N* identical particles (disks) with mass *M*. Periodic boundary conditions are applied in both directions. We study two types of particle interactions to simulate behaviors of hard disks and a DDQC-forming system, respectively.

The first interaction is harmonic repulsion. The interaction potential between particles *i* and *j* is


\begin{eqnarray*}
U( r_{ij}) = \frac{\epsilon }{2} \bigg ( 1-\frac{r_{ij}}{\sigma } \bigg ) ^2 \Theta \bigg ( 1-\frac{r_{ij}}{\sigma }\bigg ),
\end{eqnarray*}

where $\epsilon$ is the characteristic energy scale of the interaction, $r_{ij}$ is the distance between the two particles, $\sigma$ is the disk diameter and $\Theta (x)$ is the Heaviside step function. In the zero-temperature and zero-pressure limits ($T\rightarrow 0$ and $p\rightarrow 0$), particles interacting via harmonic repulsion behave like hard particles [38]. Here, we mainly show results for $p=10^{-4}$. At this pressure, the particle overlap in the resulting packings, measured by $1-\frac{r_{ij}}{\sigma }$, is as small as $8\times 10^{-5}$, indicating that the interacting particles are essentially in contact. We have verified that almost identical packing structures are obtained at even lower pressures. This confirms that a pressure of $10^{-4}$ is sufficiently low to approximate the hard-particle limit.

The second interaction potential is designed with two length scales to stabilize DDQCs [48–50]:


\begin{eqnarray*}
U(r_{ij}) = \epsilon \bigg ( \frac{\sigma }{r_{ij}} \bigg ) ^{14} + \frac{\epsilon }{2} [ 1-\tanh ( k r_{ij}-k\sigma _1) ],
\end{eqnarray*}

which is an inverse-power-law potential followed by a shoulder, where $\sigma$ and $\sigma _1$ are characteristic length scales of the core and the shoulder, respectively, and *k* sets the relative height of the shoulder. Under appropriate conditions, particles interacting via this TLS potential can self-assemble into DDQCs in thermodynamic equilibrium [48]. We use $\sigma _1=1.36\sigma$ and $k=10.0\sigma ^{-1}$, slightly different from Kryuchkov *et al.* [48], to achieve better DDQC order.

For both potentials, we set units of energy, length and mass to be $\epsilon$, $\sigma$ and *M*. Time is thus in units of $M^{1/2} \sigma \epsilon ^{-1/2}$. Temperature is in units of $\epsilon k_{\rm B}^{-1}$, where $k_{\rm B}$ is the Boltzmann constant. For the harmonic potential, we tessellate the packings into polygons by connecting particles in contact with bonds. For the TLS potential, we connect particles whose separation is smaller than $\sigma _1$ with bonds [48,49].

### Simulation methods

For systems with the harmonic potential, we obtain static particle packings at a given pressure by minimizing the enthalpy $H=U+pL^2$ via the fast inertial relaxation engine algorithm [52], where *U* is the total potential energy summed over all pairs of interacting particles. We use a constant pressure of $p=10^{-4}$.

For systems with the TLS potential, we minimize the total potential energy *U* at a given number density $\rho =NL^{-2}$ to obtain static packings. We also conduct molecular dynamic simulations in the canonical ensemble using lammps [53] to obtain the spatiotemporal evolution of the system. When the number density $\rho$ is around 0.94, the system self-assembles into a DDQC at low temperatures.

The athermal approach to form DDQC order (random tiling) for TLS disks consists of the following steps.

1. Identify centers of vacancy pairs by randomly selecting a sufficient number of particles in the initial square lattice, ensuring a minimum separation of $2\times \sqrt{2+\sqrt{3}}\approx 3.86$—the diameter of DDQC motifs.
2. Randomly assign a motif type (I or II) to each vacancy pair from step (1), with a 1:1 ratio between the two types.
3. Remove the particles according to the vacancy pairs determined in step (1) and motif types assigned in step (2).
4. Adjust the number density to $\rho = 0.94$ and perform energy minimization.

### Diffraction pattern

The diffraction pattern is characterized by the static structure factor:


\begin{eqnarray*}
S (\mathbf {q}) = \frac{1}{N} \langle \rho (\mathbf {q}) \rho (-\mathbf {q}) \rangle ,
\end{eqnarray*}

where $\rho (\mathbf {q}) = \sum _{j=1}^N {e^{{\rm i}\mathbf {q}\cdot \mathbf {r}_j}}$ is the Fourier transform of the density, $\mathbf {r}_j$ is the position of particle *j*, $\mathbf {q}$ is the wave vector satisfying periodic boundary conditions and $\langle \cdot \rangle$ denotes the ensemble average.

### Order parameter and correlation function

For a particle at $\mathbf {r}_i$, we define its *m*-fold bond-orientational order parameter as


\begin{eqnarray*}
\Psi _{m}( \mathbf {r}_i)= \frac{1}{n_{\rm b}}\sum _{l=1}^{n_{\rm b}}e^{{\rm i}m\theta (\mathbf {r}_i-\mathbf {r}_l)},
\end{eqnarray*}

where the sum is over all $n_{\rm b}$ neighbors, and $\theta (\mathbf {r}_i-\mathbf {r}_l)$ is the angle between $\mathbf {r}_i-\mathbf {r}_l$ and a reference direction. The average bond-orientation order is defined as $\chi _m = \langle |\Psi _m(\mathbf {r}_i)|^2\rangle$, where $\left\langle \cdot \right\rangle$ denotes the average over particles and configurations [27]. Here, we mainly show results for $m=4, 6$ and 12.

The correlation function of the *m*-fold bond-orientational order is defined as $G_{m}(r)= \langle \Psi _{m}^{*}( \mathbf {r}_i) \Psi _{m}( \mathbf {r}_j) \rangle ,$ where $r=|\mathbf {r}_i - \mathbf {r}_j|$ and $\langle \cdot \rangle$ denotes the average over all pairs of particles and configurations. For DDQCs, $G_{12}(r)$ is used to characterize the quasicrystalline order [27].

## Supplementary Material

nwag244_Supplemental_Files

## Data Availability

The data underlying this article are available on the GitHub website (https://github.com/SirNewtonsApple/Simply-form-DDQC).
