# Supplementary material for: Rapid and high-quality formation of dodecagonal quasicrystals and their approximants using a purely mechanical approach
Source: Natl Sci Rev. 2026 Apr 24;13(11):nwag244. doi: 10.1093/nsr/nwag244 (PMC13278518; doi:10.1093/nsr/nwag244)
Supplement: nwag244_Supplemental_Files [file nwag244_supplemental_files.zip › Supplementary data.pdf]

Supplementary information for "Rapid and high-quality formation of dodecagonal quasicrystals and their approximants using a purely mechanical approach"

Zhehua Jiang,[1,2] Jianhua Zhang,[2] Mengyuan Zhan,[2] Jiaqi Si,[2] Junchao Huang,[1] Hua Tong,[2,*] and Ning Xu[1,2,3,†]

[1]*Hefei National Research Center for Physical Sciences at the Microscale and CAS Key Laboratory of Microscale Magnetic Resonance, University of Science and Technology of China, Hefei 230026, People's Republic of China*

[2]*Department of Physics, University of Science and Technology of China, Hefei 230026, People's Republic of China*

[3]*College of Physics, Guizhou University, Guiyang 550025, People's Republic of China*

SQUARE-TRIANGLE TILING OF PERFECT DDQC

Perfect (or ideal) DDQC can be constructed using the inflation/deflation method based on squares, equilateral-triangles and rhombus [1, 2]. Simplified DDQC consists only squares and equilateral-triangles based on the modified Schlottmann tiling [3–5].

The inflation/deflation method uses self-similarity feature inherited in quasicrystals. This method [4] includes a deflation rule (or inflation rule in the same but reversed manner) and a majority rule, and starts from a hexagon consisting of 6 equilateral-triangles surrounding a vertex. The hexagon is denoted as Order 0 in Supplementary Fig. 1c, with edge length of 1. Using the deflation rule, one can replace all 6 triangles of Order 0 with the tiled triangle in Supplementary Fig. 1a, resulting in a dodecagon with edge length of $2-\sqrt{3}$, denoted as S0 in Supplementary Fig. 1c. The inner dodecagon in S0 is then replaced with a motif in Supplementary Fig. 1b. As shown in Supplementary Fig. 1b, there are two types of motifs, i.e., motifs I and II as defined in the main text. They differ orientationally by

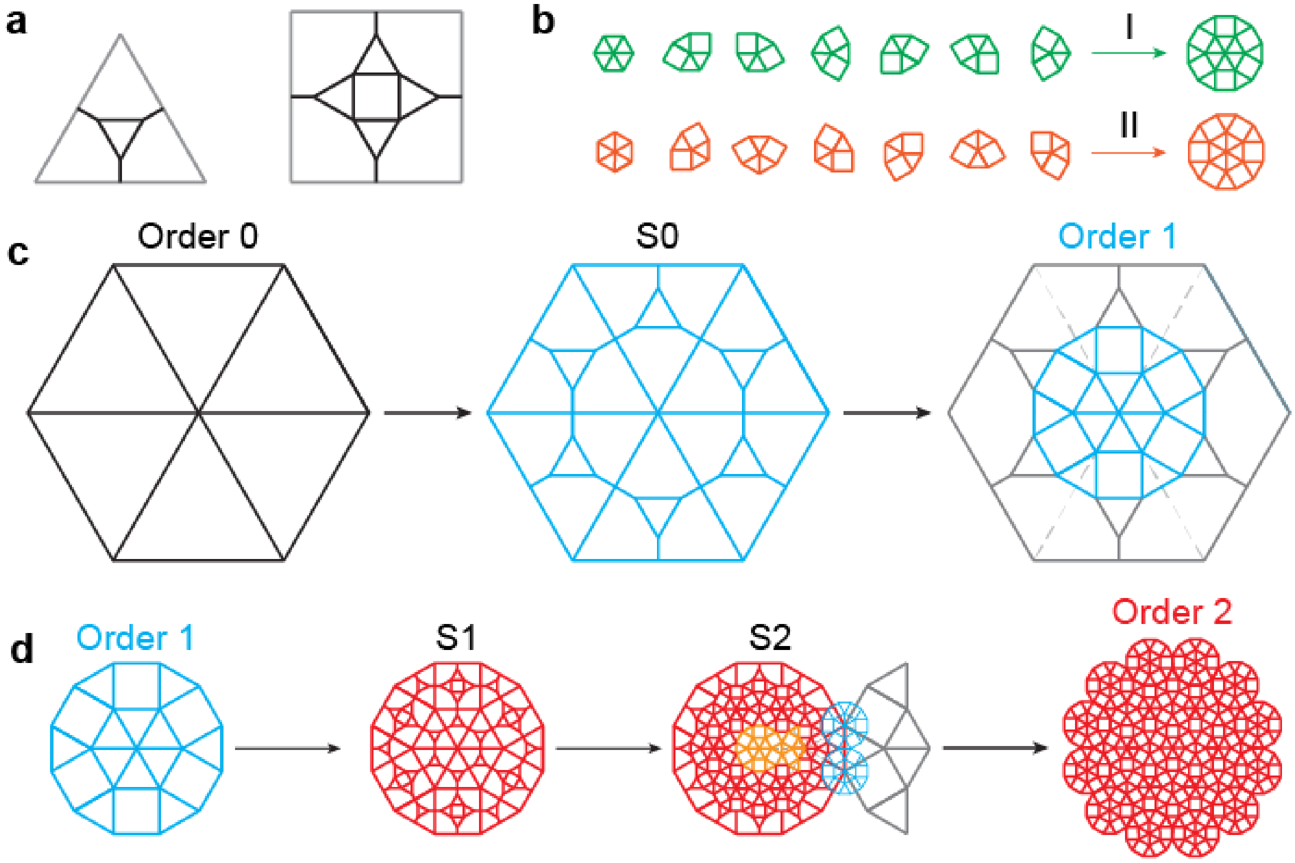

Supplementary Fig. 1. **Deflation method to construct perfect DDQC. a** Deflation rule partitions the grey triangle and square with edge length of 1 into inner black tilings with edge length of $2-\sqrt{3}$. **b** Motifs I and II and their components. **c** Construction route from Order 0 to Order 1. **d** Construction route from Order 1 to Order 2.

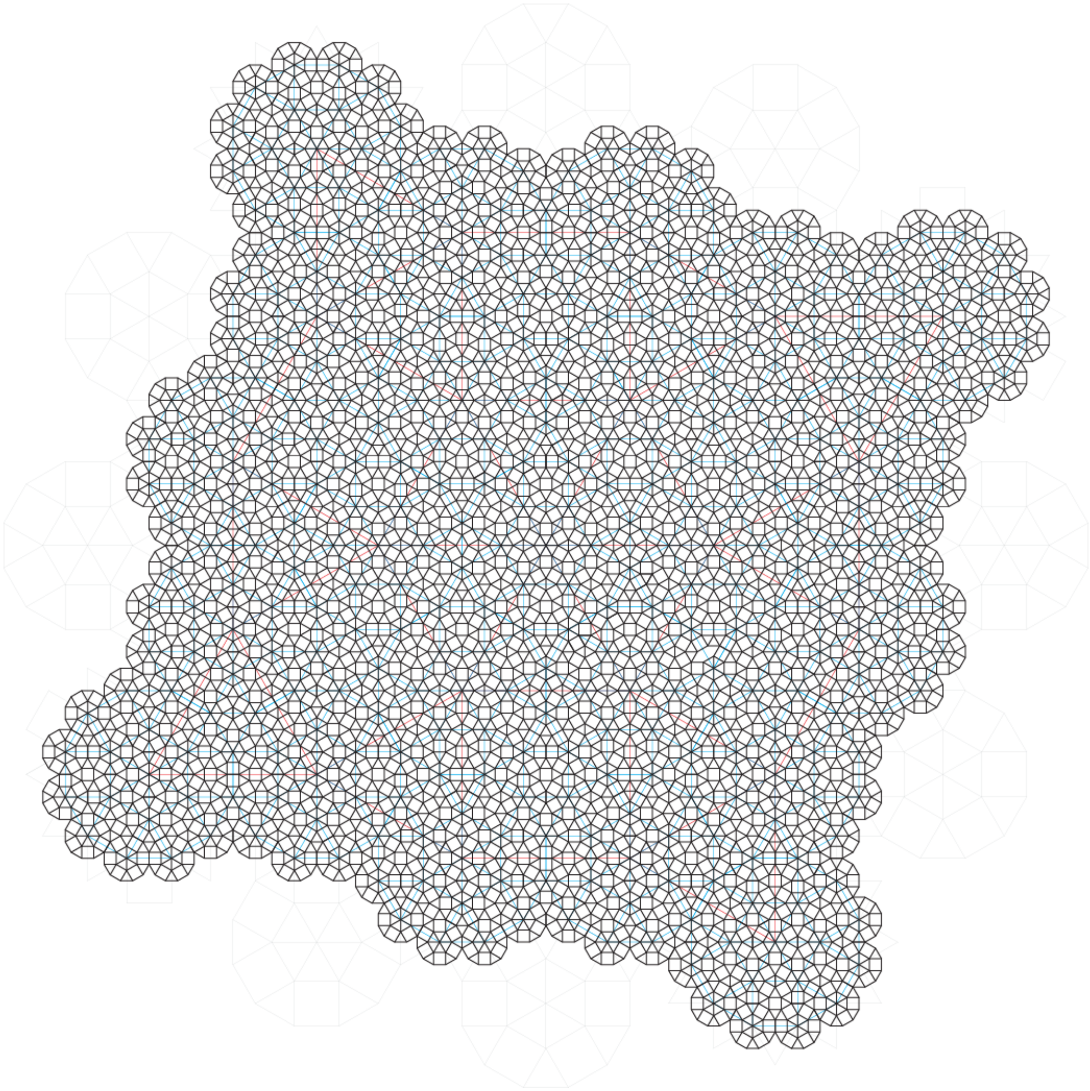

Supplementary Fig. 2. **Perfect Order 3 square-triangle DDQC tiling from the inflation/deflation method**. Order 1, Order 2, and Order 3 tilings are plotted in red, blue, and black, respectively. Light grey lines are references to determine motifs on the boundary, which are self-similar to the inner part of Order 3.

30°. The motif is chosen according to the majority rule: the 6 radial lines in the inner hexagon of the motif cover most of the lines in S0 when their centers are aligned. This chosen motif becomes Order 1 in Supplementary Fig. 1c. Note that the inner hexagon of Order 1 has exactly the same orientation as Order 0.

Supplementary Fig. 1d demonstrates how to construct a higher order tiling from Order 1. Replacing all triangles and squares in Order 1 with the tiled triangle and square in Supplementary Fig. 1a (i.e., deflation rule) leads to the tiling denoted as S1 in Supplementary Fig. 1d. Using the majority rule, all dodecagons in S1 are replaced with motifs in Supplementary Fig. 1b, leading to the tiling denoted as S2 in Supplementary Fig. 1d. Then we stick a grey tiling with the same scaling as Order 1 to S2. Note that Order 1 (parent state of S2) and the grey tiling is self-similar to the orange tiling highlighted in S2. With the aid of the grey tiling and using the deflation and majority rules, one can replace two 5/12 dodecagons on the boundary of S2 with two motifs highlighted in blue in Supplementary Fig. 1d. All 12 5/12 dodecagons on the boundary can be replaced with motifs by repeating the same process, leading to Order 2 (a larger DDQC tiling) as shown in Supplementary Fig. 1d.

In Supplementary Fig. 2, we show a perfect Order 3 square-triangle DDQC tiling constructed by the inflation/deflation method.

SMALL-AMPLITUDE OSCILLATORY SHEAR

Oscillatory shear can play a similar role as thermalization to search for more stable states [6, 7]. We apply a small-amplitude oscillatory, quasistatic shear (illustrated in Supplementary Fig. 3a) to the random tiling in Fig. 4a of the main text under the Lees-Edwards boundary conditions [8]. When the shear amplitude is small, multiple shear cycles lead to an absorbing state, i.e., a cyclic state with repeated stress-strain loop (red loop in Supplementary Fig. 3b). As shown in Supplementary Fig. 3c-f, the small-amplitude oscillatory shear optimizes the DDQC order, demonstrated by both $G_{12}(r)$ and the deviation of bond angle from the characteristic DDQC angle, $\Delta\theta$, similar to the effects induced by thermalization in the main text.

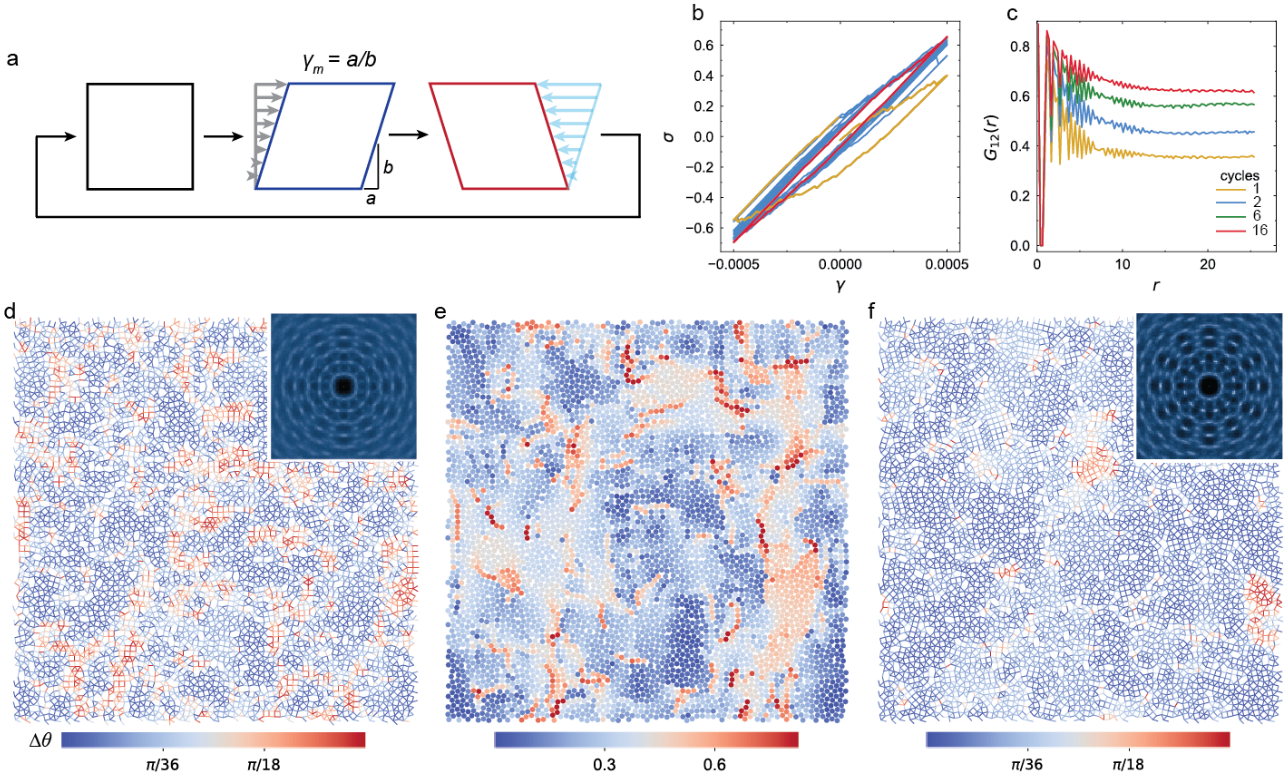

Supplementary Fig. 3. **Optimizing the DDQC order using small-amplitude oscillatory shear. a** Schematic plot of the oscillatory shear. **b** Stress-strain (σ-γ) relation during the oscillatory shear. The shear is applied to the same state in Fig. 4a of the main text. The strain amplitude is $\gamma_{\rm m} = 5 \times 10^{-4}$. The first and last cycles are drawn in orange and red, respectively. Other cycles are in blue. **c** Evolution of the correlation function $G_{12}(r)$ with shear cycles. The long-distance plateau of $G_{12}(r)$ grows with the number of cycles, indicating the growth of the DDQC order. **d-f** The same presentation as Fig. 4 of the main text, showing the effect of oscillatory shear in the optimization of the DDQC order.

DDQC APPROXIMANT IN THREE DIMENSIONS

Similar to Fig. 1 of the main text, we apply a small perturbation to a cubic packing of spheres interacting via the harmonic potential. At low pressures, the cubic packing of these hard-sphere-like particles transforms to a packing illustrated in Supplementary Fig. 4a. As shown in Supplementary Fig. 4b-d, the x-y, y-z, and z-x planes exhibit the (3^2.4.3.4) Archimedean tiling. However, the diffraction pattern in the [100] direction still shows the 4-fold crystalline symmetry (Supplementary Fig. 4e).

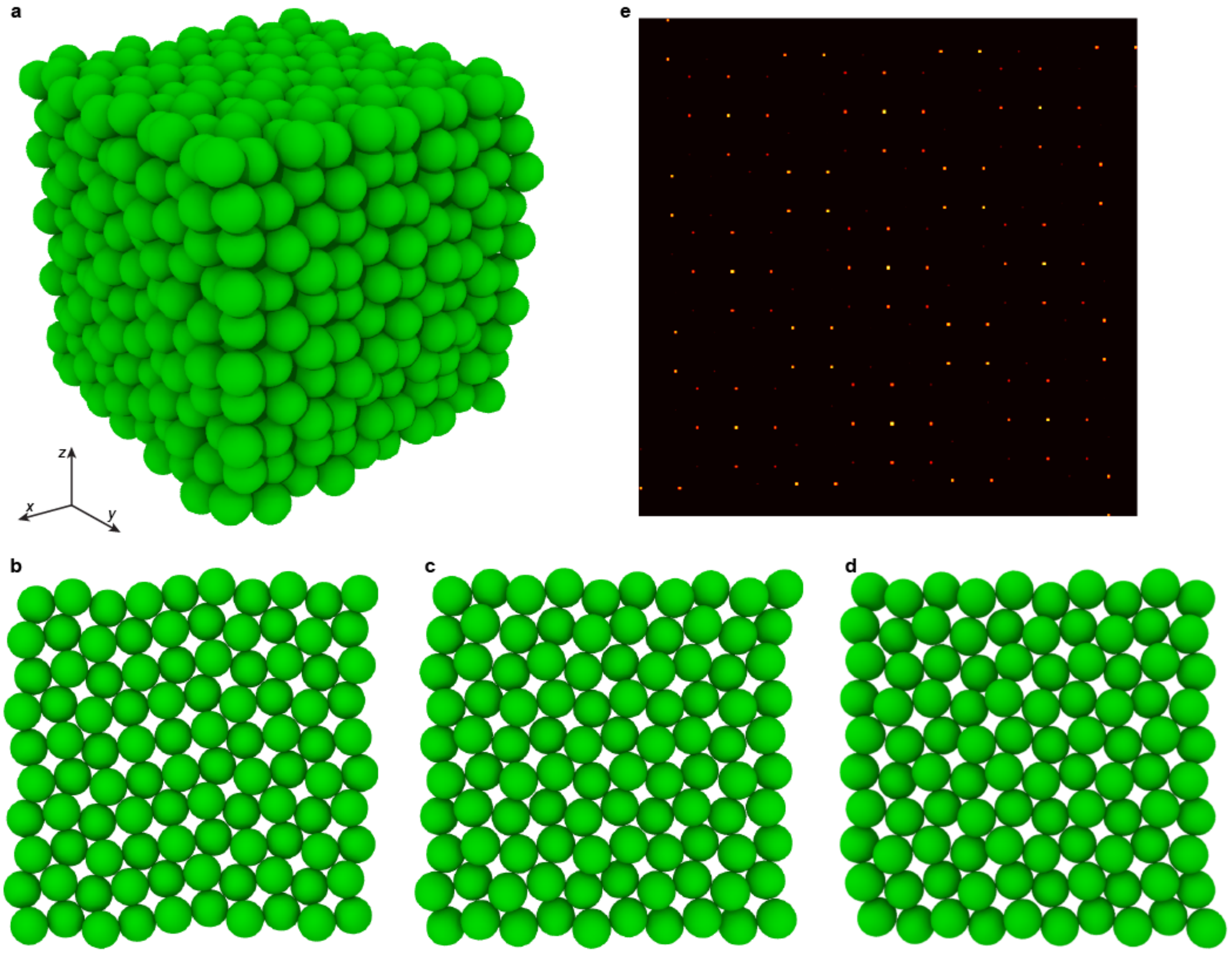

Supplementary Fig. 4. **DDQC approximant in three dimensions**. **a** Packing of spheres interacting via the harmonic potential induced by perturbing a cubic packing and minimizing the enthalpy at $p = 10^{-4}$. **b-d** Structures of the packing in the x-y, y-z, and z-x planes. **e** Diffraction pattern in the [100] direction.

TEMPERATURES FOR EFFECTIVE DDQC-ORDER ENHANCEMENT VIA THERMAL TREATMENT

To obtain DDQCs, the temperature must be maintained below the melting temperature T_{m}. For the TLS potential studied in the main text, T_{m} can be determined via the temperature dependence of the internal energy E, as illustrated in Supplementary Fig. 5a. To measure $E(T)$, we start with the near-perfect DDQC single tiling (Fig. 6b of the main text) and heat it from $T = 0$ to $T = 0.4$. During heating, the temperature is increased by $\Delta T = 0.0025$ per step, and the system is equilibrated for a time of 500 at each step in the canonical ensemble, after which the internal energy E is measured as a time average over the subsequent time of 500. The energy jump at $T_{\mathrm{m}} \approx 0.24$ in Supplementary Fig. 5a indicates the melting point. Supplementary Fig. 5a also illustrates the diffraction patterns of configurations before (at $T = 0.212$) and after (at $T = 0.265$) melting, which clearly exhibit 12-fold-symmetry order and random order, respectively.

Supplementary Fig. 5b compares the correlation function $G_{12}(r)$ at various temperatures, measured after thermalizing the mechanically prepared state for a time of 5×10^3. For $T < 0.22$ (only slightly below T_{m}), a clear enhancement of the DDQC order is observed compared to the results in Fig. 5 of the main text.

These results collectively indicate that $T_{\mathrm{m}} \approx 0.24$ sets the upper limit for thermal enhancement. As long as the temperature is significantly lower than T_{m}, the thermal enhancement of the DDQC order is effective.

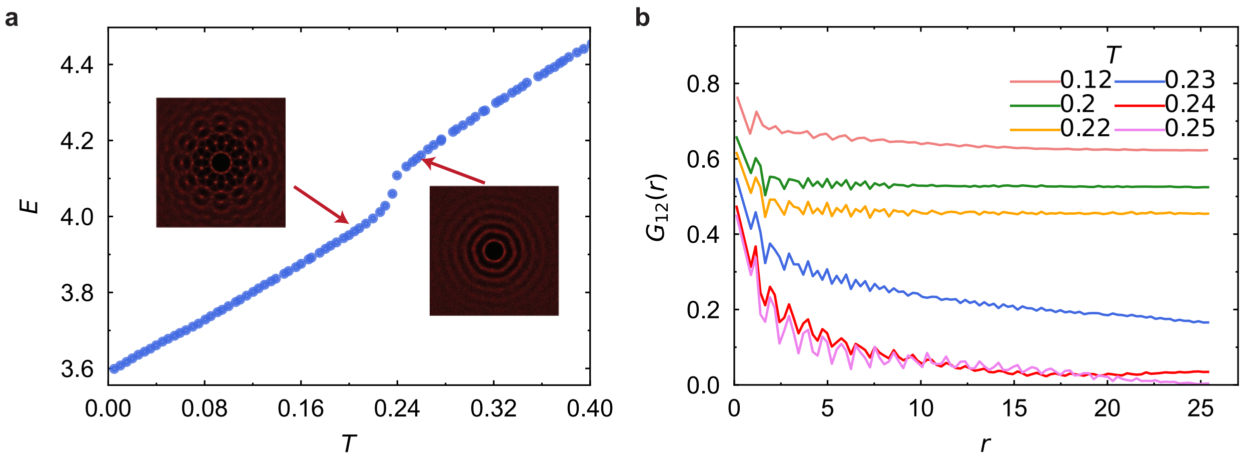

Supplementary Fig. 5. Effectiveness of thermal enhancement as a function of temperature. a Temperature dependence of the internal energy E. The red arrows indicate the temperatures ($T = 0.212$ and 0.265) for which the diffraction patterns are shown as the insets. **b** Temperature dependence of the correlation function of the 12-fold order parameter $G_{12}(r)$, measured at $t = 5 \times 10^3$. The protocol is the same as that in Fig. 5 of the main text.

FORMATION OF CONTIGUOUS DDQC MOTIFS

The DDQC motifs in the perfect tiling shown in Supplementary Fig. 2 are contiguous with each other. Achieving this specific tiling pattern is essential for forming the near-perfect, single DDQC tiling presented in Fig. 6b of the main text. The relative placement of motif centers is critical: if two centers are insufficiently separated, the induced motifs will overlap and disrupt the local DDQC order. Conversely, if the centers are sufficiently far apart, each motif can be preserved, but they will no longer be contiguous. Through numerical tests, we have verified that contiguous motifs can indeed be generated when vacancy pairs are arranged as illustrated in Supplementary Fig. 6. To realize such an arrangement globally within an initial square lattice, a specific ratio between the lattice constants is required: the lattice constant of the first-order hyper-particles must be approximately 4.1 times that of the original square lattice.

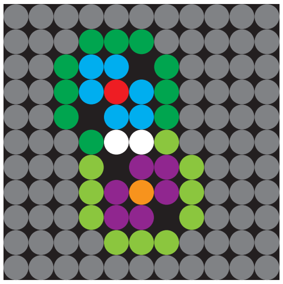

Supplementary Fig. 6. **Arrangement of two vacancy pairs for forming contiguous DDQC motifs.** Disks forming the two motifs are distinguished by color. The first motif has its center at the red circle, with first-shell disks in blue and second-shell disks in green. The second motif has its center at the orange circle, with first-shell disks in purple and second-shell disks in light green. White disks are shared by both motifs.

DEFECTS IN SQUARE-TRIANGLE TILING

A two-dimensional plane can be tiled with squares and triangles in only four Archimedean tilings: (3^6), ($3^3.4^2$), ($3^2.4.3.4$) and (4^4), as shown in Supplementary Fig. 7a. Any arrangement that deviates from these four patterns is considered as a topological point defect [9]. An example is shown in Supplementary Fig. 7b, where such point defects are marked by hollow circles.

Supplementary Fig. 8 shows another way to identify defective particles and quantitative measure the deviation from an ideal single DDQC. We overlay the square-triangle tiling of the ideal single DDQC onto the resulting packing in Fig. 6b of the main text. Most disks coincide with a tiling node. Only isolated disks (red) do not coincide with any nodes and are thus considered defective. If these defective disks were excluded, the packing would be topologically perfect. Therefore, the fraction of these defective disks serves as a quantitative measure of the deviation from an ideal single DDQC.

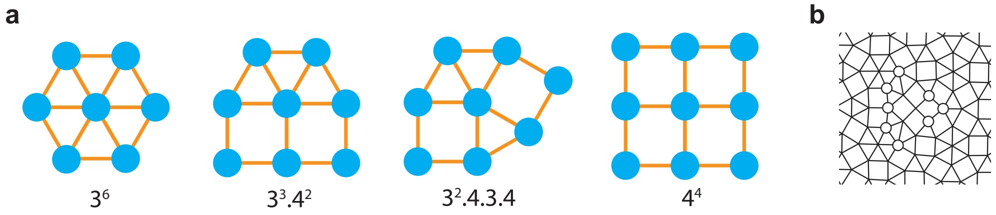

Supplementary Fig. 7. **Determination of point defects**. **a** Archimedean square-triangle tilings that can cover a two-dimensional plane. **b** An example of topological point defects (circles) in a square-triangle tiling.

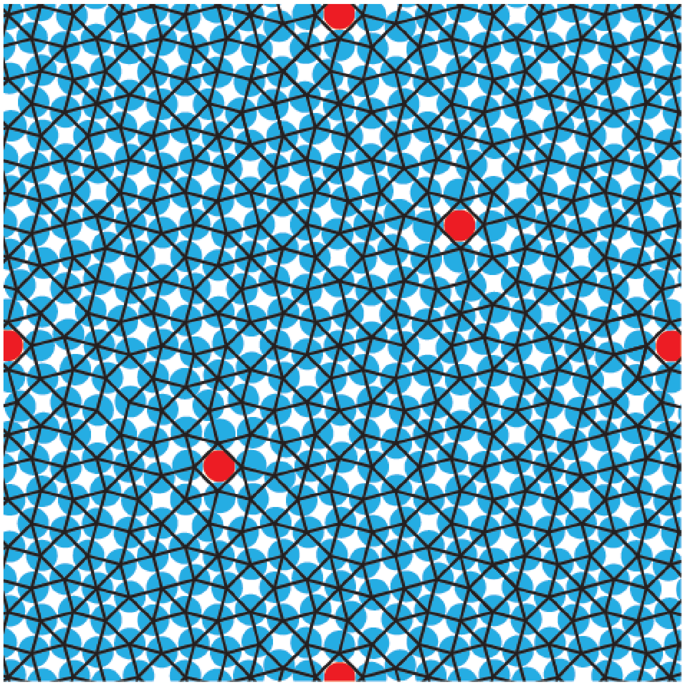

Supplementary Fig. 8. **A quantitative measure of the deviation from perfect DDQC order.** Blue circles are disks of the near-perfect tiling in Fig. 6b of the main text. Black lines represent the square-triangle tiling of the perfect DDQC. Red disks that do not coincide with any tiling nodes are considered defective.

SUBSEQUENT REMOVAL OF VACANCY PAIRS

Here we present an alternative, experimentally more feasible protocol for the mechanical approach: (1) Locate a sufficient number of vacancy pairs and assign their motif types; (2) Remove a small subset of vacancy pairs; (3) Minimize the energy and compress the system to the fixed target density; (4) Repeat steps (2) and (3) until all vacancy pairs are removed; (5) Equilibrate the configuration in the canonical ensemble. This stepwise procedure more accurately reflects experimentally feasible conditions, under which only a limited number of particles are removed at a time.

We perform a test by starting with a square lattice of $N = 6400$ TLS particles and removing one vacancy pair per step until a total of $n_\mathrm{v} = 427$ vacancy pairs have been removed. The target number density is still $\rho = 0.94$. The corresponding results are presented in Supplementary Fig. 9. As more vacancy pairs are removed, the configuration exhibits better 12-fold symmetry (Supplementary Fig. 9a), similar to that observed in Fig. 3c of the main text, where all vacancy pairs are removed simultaneously.

Supplementary Fig. 9b and c show that the DDQC order is established after $n_\mathrm{v} = 427$ vacancy pairs are removed and is further enhanced by thermal treatment at $T = 0.12$ for a duration of $t = 5 \times 10^5$, respectively. Comparing Supplementary Fig. 9b and c, the bond-angle deviation $\Delta\theta$ decreases globally upon thermal treatment. Moreover, the diffraction pattern becomes sharper, reflecting a clearer 12-fold DDQC order after annealing.

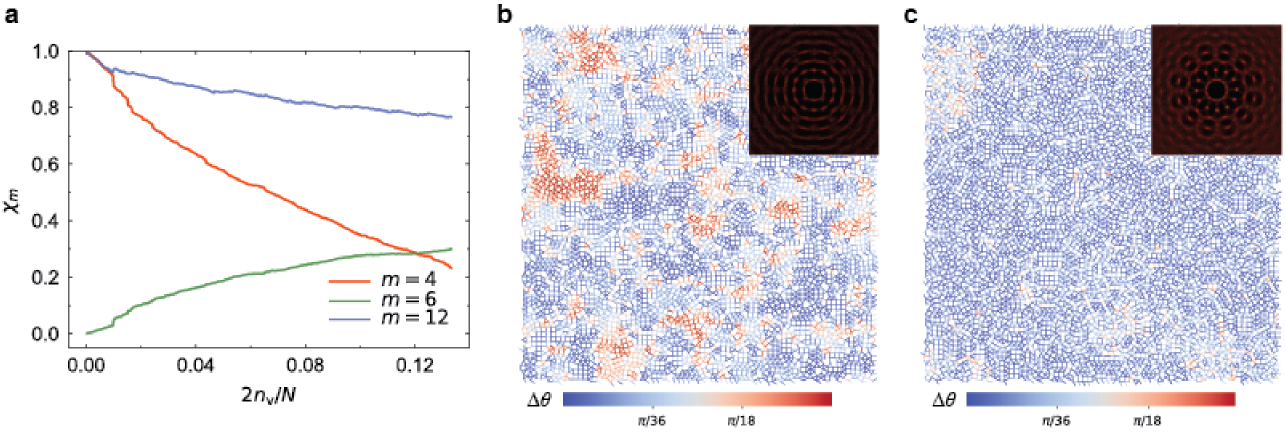

Supplementary Fig. 9. **Subsequent removal of vacancy pairs.** **a** Evolution of m-fold order parameter χ_m for subsequent removal of vacancy pairs. **b** Spatial distribution of $\Delta\theta$ for a configuration after subsequent removal of $n_\mathrm{v} = 427$ vacancy pairs. **c** Spatial distribution of $\Delta\theta$ after equilibrating the configuration in **b** with a duration of $t = 5 \times 10^5$ at $T = 0.12$. Diffraction pattern for each configuration is inserted in **b,c**.

PARTICLE REMOVAL IN HEXAGONAL LATTICE

In this study, our primary results were obtained by removing vacancy pairs from an initial square lattice. It is natural to ask whether other initial packings, such as the hexagonal lattice, can also yield DDQC order. Supplementary Fig. 10 shows four vacancy-removal schemes in a hexagonal lattice; None of them succeeds in generating local DDQC order. Therefore, the special square-DDQC transformation pathway shown in Fig. 1c and Fig. 2a-b of the main text is crucial for establishing local DDQC order.

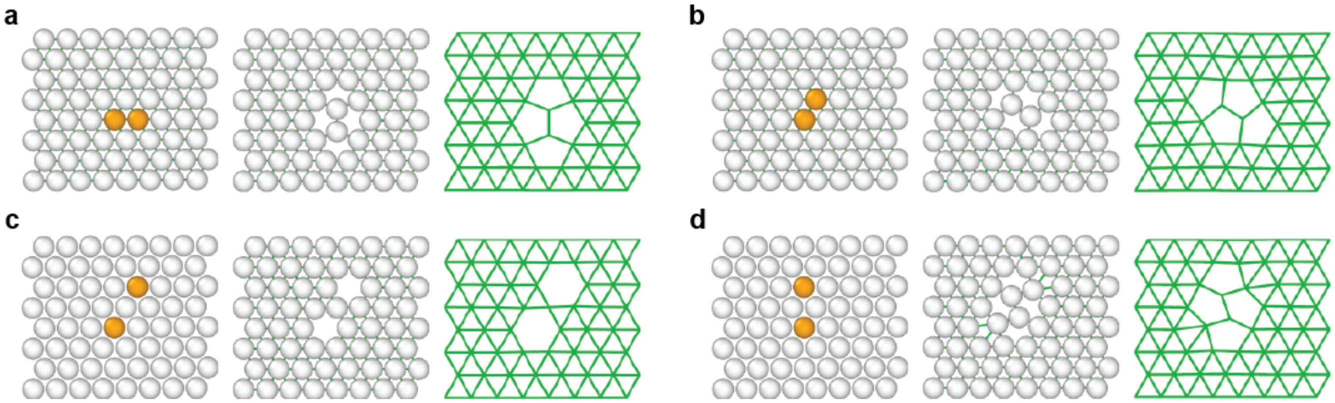

Supplementary Fig. 10. **Particle-removal schemes in a hexagonal lattice. a-d** Four vacancy-pair-removal schemes. For each scheme, the left panel shows the initial hexagonal packing, with particles to be removed highlighted in orange. After particle removal, energy minimization is performed at a density of $\rho = 0.94$, yielding the configuration shown in the middle panel. The corresponding bond structure is displayed in the right panel.

PREDICTING DDQC SELF-ASSEMBLY

The mechanical approach leads to rapid formation of DDQCs under DDQC-forming conditions. Furthermore, our study proposes a method for predicting whether a system can self-assemble into a DDQC under other conditions. As illustrated in Supplementary Fig. 11, at densities where DDQCs are thermodynamically unstable, our mechanical approach would not yield any DDQC structures. Therefore, the emergence of DDQC structures via our mechanical approach may serve as an indicator of the DDQC-forming capability.

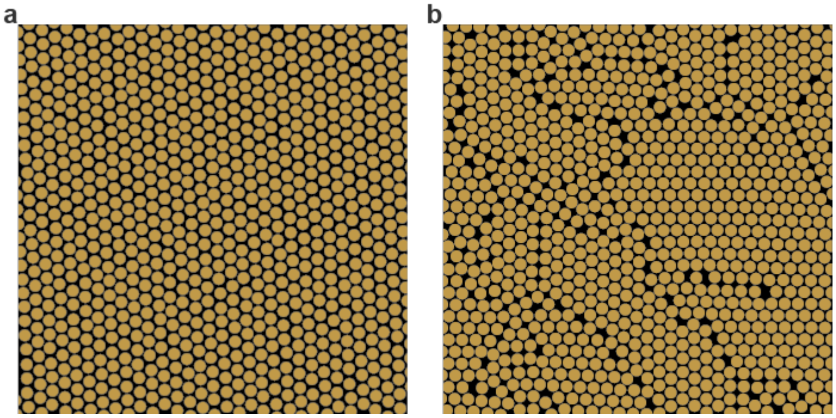

Supplementary Fig. 11. Example of the failure of the mechanical approach to induce quasicrystalline order in a non-DDQC-forming system. (a) A state of TLS disks after thermal equilibration of an $N = 900$ liquid at $T = 0.12$ and $\rho = 1.02$. The equilibrium state is the hexagonal phase. (b) A packing of the same system in (a) generated by introducing $n_{\rm v} = 70$ vacancy pairs to an $N = 1089$ square packing and minimizing the energy. The mechanical approach results in a polycrystalline state of the hexagonal phase rather than DDQC structures.

* Email:huatong@ustc.edu.cn
† Email:ningxu@ustc.edu.cn

[1] Stampfli P. A dodecagonal quasiperiodic lattice in two dimensions. *Helv Phys Acta* 1986; **59**: 1260–3.
[2] Gähler F. Crystallography of dodecagonal quasicrystals. In: Janot C and Dubois JM (ed.). *Quasicrystalline materials: Proceedings of the ILL/CODEST Workshop*. Singapore: World Scientific, 1988, 272–84.
[3] Hermisson J, Richard C, Baake M. A guide to the symmetry structure of quasiperiodic tiling classes. *J Phys I France* 1997; **7**: 1003–18.
[4] Sadoc J and Mosseri R. Quasiperiodic Frank–Kasper phases derived from the square–triangle dodecagonal tiling. *Struct Chem* 2017; **28**: 63–73.
[5] Impéror-Clerc M, Jagannathan A, Kalugin P *et al.* Square-triangle tilings: an infinite playground for soft matter. *Soft Matter* 2021; **17**: 9560–75.
[6] Regev I, Lookman T, Reichhardt C. Onset of irreversibility and chaos in amorphous solids under periodic shear. *Phys Rev E* 2013; **88**: 062401.
[7] Fiocco D, Foffi G, Sastry S. Oscillatory athermal quasistatic deformation of a model glass. *Phys Rev E* 2013; **88**: 020301.
[8] Maloney CE and Lemaître A. Amorphous systems in athermal, quasistatic shear. *Phys Rev E* 2006; **74**: 016118.
[9] Leung PW, Henley CL, Chester GV. Dodecagonal order in a two-dimensional Lennard-Jones system. *Phys Rev B* 1989; **39**: 446–58.
